# Proteomic profiling of canine fibrosarcoma and adjacent peritumoral tissue

**DOI:** 10.1016/j.neo.2022.100858

**Published:** 2022-12-09

**Authors:** Erin Beebe, Amiskwia Pöschel, Laura Kunz, Witold Wolski, Zahra Motamed, Daniela Meier, Franco Guscetti, Mirja C. Nolff, Enni Markkanen

**Affiliations:** a Institute of Veterinary Pharmacology and Toxicology, Vetsuisse Faculty, University of Zurich, 8057 Zürich, Switzerland; b Functional Genomics Center Zürich, ETH Zürich/University of Zurich, 8057 Zürich, Switzerland; c Zyto/Histo Diagnostik Labor Freienstein, 8427 Freienstein, Switzerland; d Institute of Veterinary Pathology Vetsuisse Faculty, University of Zurich, 8057 Zürich, Switzerland; e Small Animal Surgery, Tierspital Zürich, 8057 Zürich, Switzerland

**Keywords:** Soft-tissue sarcoma, Comparative oncology, Human fibrosarcoma, Laser-capture microdissection, Fibrosarcoma characterization, dog fibrosarcoma, rare cancers, adult-type fibrosarcoma not otherwise specified

## Abstract

Fibrosarcoma (FSA) are rare soft tissue tumors that display aggressive local behavior and invasive growth leading to high rates of tumor recurrence. While the low incidence in humans hampers detailed understanding of the disease, FSA are frequent in dogs and present potential models for the human condition. However, a lack of in-depth molecular characterization of FSA and unaffected peritumoral tissue (PTT) in both species impedes the translational potential of dogs. To address this shortcoming, we characterized canine FSA and matched skeletal muscle, adipose and connective tissue using laser-capture microdissection (LCM) and LC-MS/MS in 30 formalin-fixed paraffin embedded (FFPE) specimens. Principal component analysis of 3’530 different proteins detected across all samples clearly separates the four tissues, with several targets strongly differentiating tumor from all three PTTs. 25 proteins were exclusively found in tumor tissue in ≥80% of cases. Among these, CD68 (a macrophage marker), Optineurin (OPTN), Nuclear receptor coactivator 5 (NCOA5), RAP1GDS1 (Rap1 GTPase-GDP dissociation stimulator 1) and Stromal cell derived factor 2 like 1 (SDF2L1) were present in ≥90% of FSA. Protein expression across all FSA was highly homogeneous and characterized by MYC and TP53 signaling, hyperactive EIF2 and immune-related changes as well as strongly decreased oxidative phosphorylation and oxidative lipid metabolism. Finally, we demonstrate significant molecular homology between canine FSA and human soft-tissue sarcomas, emphasizing the relevance of studying canine FSA as a model for human FSA. In conclusion, we provide the first detailed overview of proteomic changes in FSA and surrounding PTT with relevance for the human disease.

## Introduction

Fibrosarcoma (FSA) are tumors of the soft tissue that affect both humans and dogs [1,2]. In both species, FSA display aggressive local behavior with diffuse tissue invasion and are characterized by low sensitivity towards radio- and chemotherapy as well as high rates of tumor recurrence [3,4]. In humans, ‘adult-type FSA not otherwise specified’ are exceedingly rare tumors (crude incidence 0,2:100.000 [5]) that are diagnosed by exclusion of other subtypes and are classified as highly malignant with a poor prognosis [3,6]. The low incidence of FSA in humans causes unique challenges for affected patients, ranging from a lack of understanding of disease mechanisms, poor diagnostic accuracy, to unsatisfactory therapy [5,7]. Setup of clinical trials to specifically address adult FSA is difficult if not impossible due to a lack of statistical power caused by small sample sizes [5,7]. Accordingly, for FSA, both its developmental origin as well as the molecular events driving the disease remain greatly understudied to date [8].

Naturally occurring tumors in pet dogs are progressively leveraged to better understand tumor biology and find novel anti-cancer treatments [9], [10], [11]. Interestingly, in contrast to humans, FSA occur much more frequently in dogs (5:100.000 [11]), rendering the dog especially valuable for comparative investigation of this tumor type. Moreover, as structured assessment of novel therapies in humans is difficult due to rarity of the disease, identification of molecular homology in canine FSA could facilitate preclinical studies and development of novel targeted therapeutic strategies for this tumor entity. As such, comparative study of FSA in dogs represents a potentially highly valuable model to better understand human FSA and to perform clinical testing of novel therapeutic modalities in.

Although it is widely accepted that FSA of dogs and humans share morphological traits and exhibit comparable clinical behavior, both human and canine FSA lack detailed molecular characterization. Hence it remains completely unclear what changes occur in tumor cells from canine FSA and how canine and human FSA compare on a molecular level. Moreover, it remains unknown what differentiates FSA from the unaffected surrounding peritumoral tissue (PTT) in either species. This striking lack of data on an important tumor type prevents unbiased cross-species analysis of molecular homology and difference, therefore undermining the validity of the canine model for human disease. More detailed insight into the molecular landscape of these tumors is urgently needed to enable development of better treatment modalities for patients suffering from FSA.

We have established a powerful approach to isolate microscopically defined tissue areas from formalin-fixed paraffin embedded (FFPE) clinical tumor samples using laser-capture microdissection (LCM) followed by LC-MS/MS [12], [13], [14], [15], [16], [17]. Here, we apply this approach to analyze tumor and matched PTT from 30 dogs using LC-MS/MS to gain detailed molecular insight into the proteomic changes of spontaneous canine FSA.

## Material and methods

### Ethics approval and consent to participate

No animals were killed for the purpose of this research project, as the tissue analysed had been surgically removed in a curative setting with the verbal consent of the patient owners. According to the Swiss Animal Welfare Law Art. 3 c, Abs. 4 the preparation of tissues in the context of agricultural production, diagnostic or curative operations on the animal or for determining the health status of animal populations is not considered an animal experiment and, thus, does not require an animal experimentation license. The use of FFPE material from canine patients which was obtained for diagnostic reasons therefore does not require a formal ethics approval and complies with national guidelines.

### Selection of cases for LCM

Fibrosarcoma and matched adjacent PTT (skeletal muscle (SM), adipose tissue (AT) and connective tissue (CT)) were concurrently isolated using laser-capture microdissection from FFPE tissue of 30 canine FSA samples that were provided by the Institute of Veterinary Pathology of the Vetsuisse Faculty Zürich. All samples were FFPE samples either from the Small Animal Hospital of Zurich or external cases sent in by veterinarians practicing in Switzerland. Cases were selected by a certified pathologist (FG) according to criteria indicated by [18]. Paraffin blocks were routinely kept at room temperature. Tissue processing for LCM was performed as previously described [14] with an updated staining protocol (Supplementary Table 3). All cases were reviewed by a veterinary pathologist (FG). Table 1 provides clinical details, such as age and breed of each patient, sample age and tumor type, for all cases included in the study.

**Table 1:** **Overview of cases included in this study.** f = female, f/n = female neutered, m = male, m/n = male neutered, age = age of patient at excision of tumor. The last three columns indicate peritumoral tissues collected for the respective cases, where AT = adipose tissue, CT = connective tissue, SM = skeletal muscle tissue.

Case ID	Breed	Age (y)	Sex	Anatomical location	AT	CT	SM
1	Dobermann	4	f/n	Skin back	x	x	x
2	Bernese Mountain Dog	10	F	Skin back	x	x	x
3	Great Dane	3	m/n	Skin knee	x	x	
4	Rhodesian Ridgeback	6	f/n	Skin flank	x	x	x
5	German Shepherd	8	f/n	Skin	x	x	x
6	Rhodesian Ridgeback	2	F	Skin shoulder	x	x	
7	Irish Setter	6	M	Skin shoulder	x	x	
8	Boxer	7	M	Skin chest	x	x	
9	Vizsla	8	M	Skin back	x	x	x
10	American Staffordshire mixed	6	M	Skin flank	x	x	x
11	Old English Bulldog	5	f/n	Skin thigh	x	x	x
12	Golden Retriever	3	M	Skin neck	x	x	x
13	Bichon Frisé	11	f/n	Skin forehead	x	x	x
14	Beagle	12	M	Skin jaw		x	x
15	Irish Setter	13	f/n	Skin head			x
16	Pug	6	Fn	Skin head		x	
17	Mixed breed	5	M	Skin paw	x		x
18	Briard	5	m/n	Skin nape			x
19	Akita Inu	2	M	Skin thigh	x	x	
20	Appenzeller Sennenhund	6	m/n	Skin leg		x	
21	Labrador Retriever	4	f/n	Skin paw		x	
22	Poodle	8	f/n	Skin flank		x	
23	Mixed breed	7	m/n	Skin knee			
24	Jack Russel Terrier	6	f/n	Skin nape	x	x	
25	Hovawart	12	M	Skin chest		x	
26	German Pinscher	9	f/n	Skin head		x	x
27	Golden Retriever	11	M	Skin chest			x
28	Gordon Setter	9	f/n	Skin flank		x	x
29	Labrador Retriever	11	m/n	Skin flank	x	x	x
30	Mixed breed	11	f/n	Skin head		x	

### Laser-capture microdissection (LCM)

Laser-capture microdissection was performed using the ArcturusXT^TM^ Laser Capture Microdissection System (Thermo Scientific) as described (Amini et al., 2017). Areas of interest were identified and isolated according to the manufacturer's protocol and the criteria described in [12,13]. Isolation of areas of interest was verified by microscopic examination of the LCM cap as well as the excised region after microdissection. 2 caps were collected per case and tissue. After excision, the caps containing tissue were put on a 1.5 ml centrifuge tube (EppendorfⓇSafe-Lock tubes) and frozen at −20°C until further processing.

### Sample preparation for proteomic analysis

For protein extraction, sterile blades and forceps were used to peel off the thermoplastic membranes containing captured cells from the cap, which were then transferred into a sterile Eppendorf^Ⓡ^Safe-Lock tube. The rehydration of the laser capture microdissected tissue was done by adding 900 µl of heptane and incubating for 10 min at 30°C in a thermomixer (800 rpm). After centrifugation (20000 x g, 10 min), the heptane was removed and this step was repeated. In addition, the membranes were washed with 900 µl of ethanol (5 min, RT, 1000 rpm), 200 µl of 90% ethanol (5 min, RT, 1000 rpm) and 200 µl of 75% ethanol (5 min, RT, 1000 rpm). The samples were stored at -80°C overnight.

The samples were then prepared by using a commercial iST Kit (PreOmics, Germany) with an updated version of the protocol. Briefly, the tissues were solubilized in ‘Lyse’ buffer, boiled at 95°C for 60 minutes and processed with High Intensity Focused Ultrasound (HIFU) for 2 times 60 seconds setting the ultrasonic amplitude to 85%. After 1:1 dilution with water, the protein concentration was estimated using the Qubit® Protein Assay Kit (Life Technologies, Zurich, Switzerland. Afterwards, the samples were transferred to the cartridge and digested by adding 50 µl of the ‘Digest’ solution. After 3 hours of incubation at 37°C the digestion was stopped with 100 µl of Stop solution. The solutions in the cartridge were removed by centrifugation at 3800 x g, while the peptides were retained by the iST-filter. Finally, the peptides were washed, eluted, dried and re-solubilized in 20 µL of MS-solution (3% acetonitrile, 0.1% formic acid) for LC-MS-Analysis.

### Liquid chromatography-mass spectrometry analysis

Mass spectrometry analysis was performed on an Orbitrap Fusion Lumos mass spectrometer (Thermo Scientific) equipped with a Digital PicoView source (New Objective) and coupled to a M-Class UPLC (Waters). Solvent composition at the two channels was 0.1% formic acid for channel A and 0.1% formic acid, 99.9% acetonitrile for channel B. For each sample 2 µl of peptides were loaded on a commercial MZ Symmetry C18 Trap Column (100 Å, 5 µm, 180 µm × 20 mm, Waters) followed by nanoEase MZ C18 HSS T3 Column (100 Å, 1.8 µm, 75 µm × 250 mm, Waters). The peptides were eluted at a flow rate of 300 nL/min. After an initial hold at 5% B for 3 min, a gradient from 5 to 24% B in 83 min and to 36% B in 10 min was applied. The column was washed with 95% B for 10 min and afterwards the column was re-equilibrated to starting conditions for additional 10 min.

Samples were acquired in a randomized order. The mass spectrometer was operated in data-independent mode (DIA) with a maximum cycle time of 3 s, using Xcalibur, with spray voltage set to 2.3 kV, funnel RF level at 40 %, and heated capillary temperature at 320 °C. Full-scan MS spectra (350−1’500 m/z) were acquired at a resolution of 120’000 at 200 m/z after accumulation to a target value of 200’000 or for a maximum injection time of 100 ms. DIA scans covered a range from 400 to 1000 m/z in windows of 16 m/z. The resolution of the DIA windows was set to 30’000, with an AGC target value of 50’000, the maximum injection time set to 50 ms and a fixed normalized collision energy (NCE) of 33%. Each instrument cycle was completed by a full MS scan. The mass spectrometry proteomics data were handled using the local laboratory information management system (LIMS) [19].

### Data processing

The acquired raw MS data were processed by Spectronaut (version 14.9.201124.47784), using the directDIA analysis type. Spectra were searched against a Uniprot Canis lupus familiaris reference proteome (taxonomy 9615, canonical version from 2019-07-29). Carbamidomethylation of cysteine was set as fixed, while methionine oxidation and N-terminal protein acetylation were set as variable modifications. Enzyme specificity was set to trypsin/P, allowing a minimal peptide length of 7 amino acids and a maximum of two missed cleavages. We used Spectronaut version 14 Biognosis Factory Settings (BFS) for peptide and protein identification and quantification. Using the untransformed protein intensities reported by Spectronaut, we computed various statistics per protein and tissue, including number: of quantifications, standard deviation, mean intensity, CV, the maximum, and the minimum number of peptides identified in a single sample.

### Differential expression analysis

We performed differential protein expression analysis using the r-package prolfqua [20]. The intensities were first log2 transformed and then z-transformed so that the sample mean and variance were equal. Next, we fitted a linear model with a single factor (tissue) to each protein, and tissue differences (protein log2 fold changes) were estimated and tested using the model parameters. To increase the statistical power, we moderated the variance estimates using the empirical Bayes approach, which exploits the parallel structure of the high throughput experiment [21]. Finally, the p-values are adjusted using the Benjamini and Hochberg procedure to obtain the false discovery rate (FDR).

For the gene set enrichment analysis and KEGG pathway analysis, the tool WebGestalt (http://www.webgestalt.org) was used. Additional pathway analysis was performed with the help of QIAGEN Ingenuity Pathway Analysis (QIAGEN Inc., https://digitalinsights.qiagen.com/IPA). For the comparison of the transcriptomic and proteomic data set, the online tool Shiny App (http://fgcz-shiny.uzh.ch/fgcz_multiOmicsAnalysis_app/) run by the Functional Genomics Center Zurich, was used. Uniprot protein identifiers were first converted to ensembl and then gene names. Dog genes (CanFam3.1) were converted to human orthologues using Ensembl BioMart (release 100) prior to analysis with MetaCore [22]. For the pathway analysis, the web tool MetaCore from Clarivate Analytics™ was used (https://portal.genego.com).

### 3D Principal component analysis

Principle component analysis was performed applying prcomp on normalized protein intensity values. 3D visualization was achieved using R package scatterplot3d [23], with PC1, PC2 and PC3 as x,y and z axis values respectively.

### Venn diagram

Venn diagrams were produced using VennDiagram R package [24] with either draw.quad.venn or draw.triple.venn functions. Identification of tumor and PTT-specific proteins (Fig. 3C) was performed by separating data according to tissue group and filtering by row mean !=0 to ensure presence in at least one sample. The intersection of each tissue group was used to calculate overlapping proteins and separate tissue-specific targets.

### Heatmap

Heatmap was generated using R package ComplexHeatmap [25] with row clustering distance was set to “Euclidean” and RowAnnotation according to overall high, mid and low expression. Hallmark and pathway analysis of high and lowly expressed proteins was performed with molecular signatures database (MSigDB) [26].

### Barcodeplot

Cross-species comparative analysis of tumour-specific expression was performed using the barcode enrichment plot from limma [27]. External datasets of human sarcoma included TCGA-SARC (http://cancergenome.nih.gov/.), PXD019719 [28], GSE21122 [33] and GSE21050 [34]. All target identifiers from external datasets were summarized at the gene level using BioMart [29]. GDCquery from package TCGAbiolinks [30] was used to obtain primary tumour RNAseq expression data for the TCGA-SARC cohort. Raw data from all datasets was log2 normalised and genes were ranked according to their mean expression across all samples. Ranked position indicates expression in canine cohort (x-axis) and external human dataset (line extension of the y-axis). Only common genes in canine and human were included in the barcodeplot analysis. Pearson correlation analysis of ranked position was used to confirm significance. The top 100 common highly expressed genes from each plot were identified as the leading edge and selected for input in the venn diagram.

### Graphical display of results

GraphPad Prism, Shiny App and MetaCore were used for visual representation of the results, along with selected R packages as previously mentioned.

## Results

### Proteomic profiling of canine FSA and PTT isolated using laser-capture microdissection of FFPE samples

**Fig. 1:**
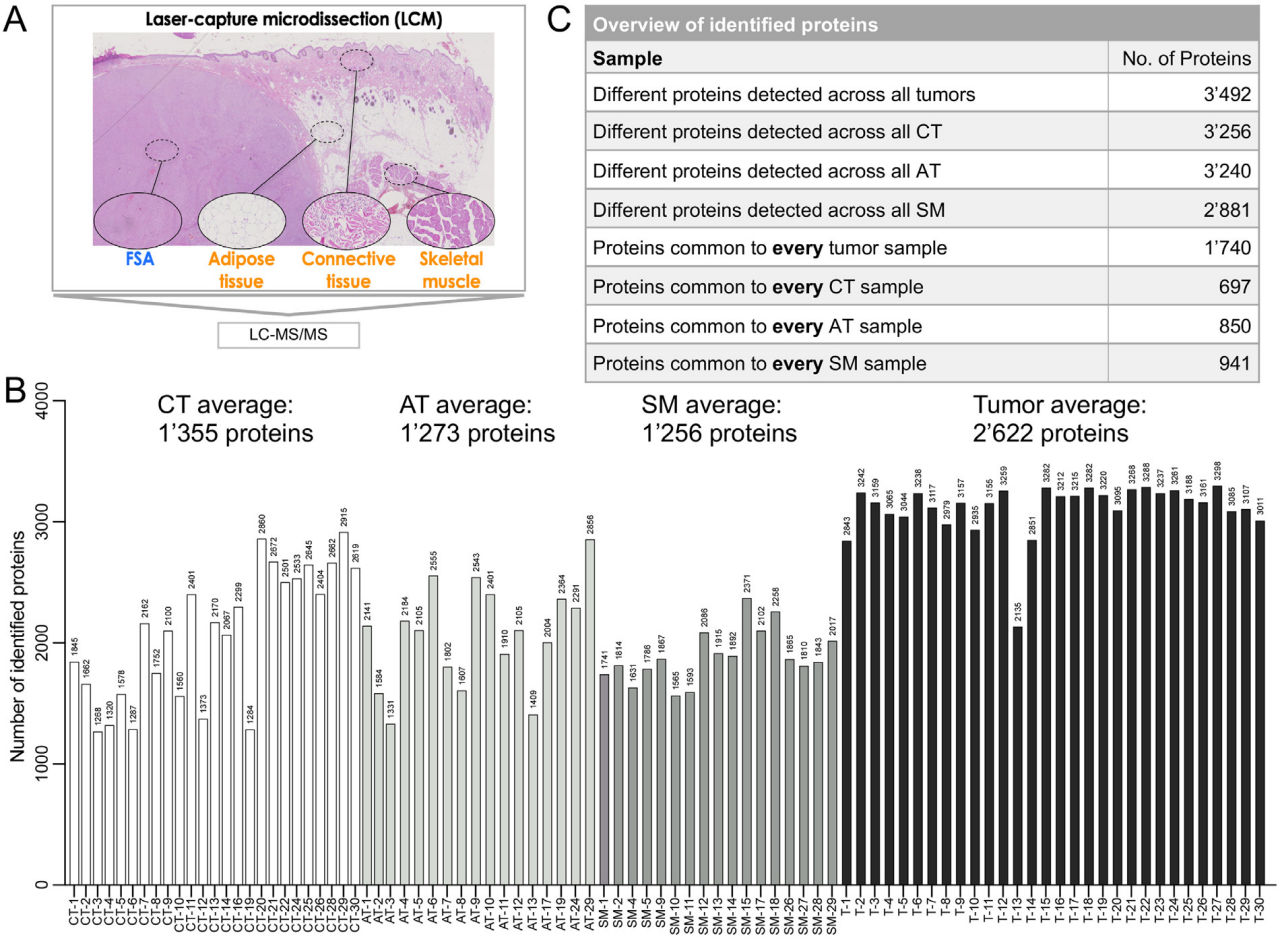
Overview of number of proteins identified in FSA and surrounding peritumoral tissue of 30 canine fibrosarcoma. A) Experimental approach to isolate FSA, adipose tissue, connective tissue and skeletal muscle from FFPE sections using laser-capture microdissection, followed by LC-MS/MS. B) Number of identified proteins (with ≥ 2 peptides/protein) for each tissue. Labels: CT = connective tissue, AT = adipose tissue, SM = skeletal muscle, T = tumor tissue, followed by the case number. C) Overview of number of identified proteins per tissue subtype.

To gain insight into the molecular features of canine FSA, we concurrently isolated tumor cells from FFPE tissue of 30 canine FSA along with matched PTT (skeletal muscle (SM) from 17 cases, adipose tissue (AT) from 17 cases and connective tissue (CT) from 25 cases, Table 1) using LCM and analyzed these specimens by LC-MS/MS (Fig. 1A). Considering only targets for which 2 or more peptides were identified, in total 3’530 different proteins could be detected across all samples, with an average of 2’622 proteins in tumor, 1’256 in SM, 1’355 in CT and 1’273 in AT (Fig. 1B and Supplementary Table 1). In general, more proteins could be detected in tumor tissue than in the surrounding PTT (Fig. 1B). In tumor tissue we found a total of 3’492 different proteins, of which 1’740 were common to every tumor sample (Fig. 1C). Across all CT samples 3’256 different proteins were identified, of which 697 proteins could be detected in every CT specimen. 3’240 different targets were present in AT, and 850 of these were common to all AT samples. And for SM, we identified 2’881 different proteins in total, with 941 of them found in every SM tissue (Fig. 1C).

**Fig. 2:**
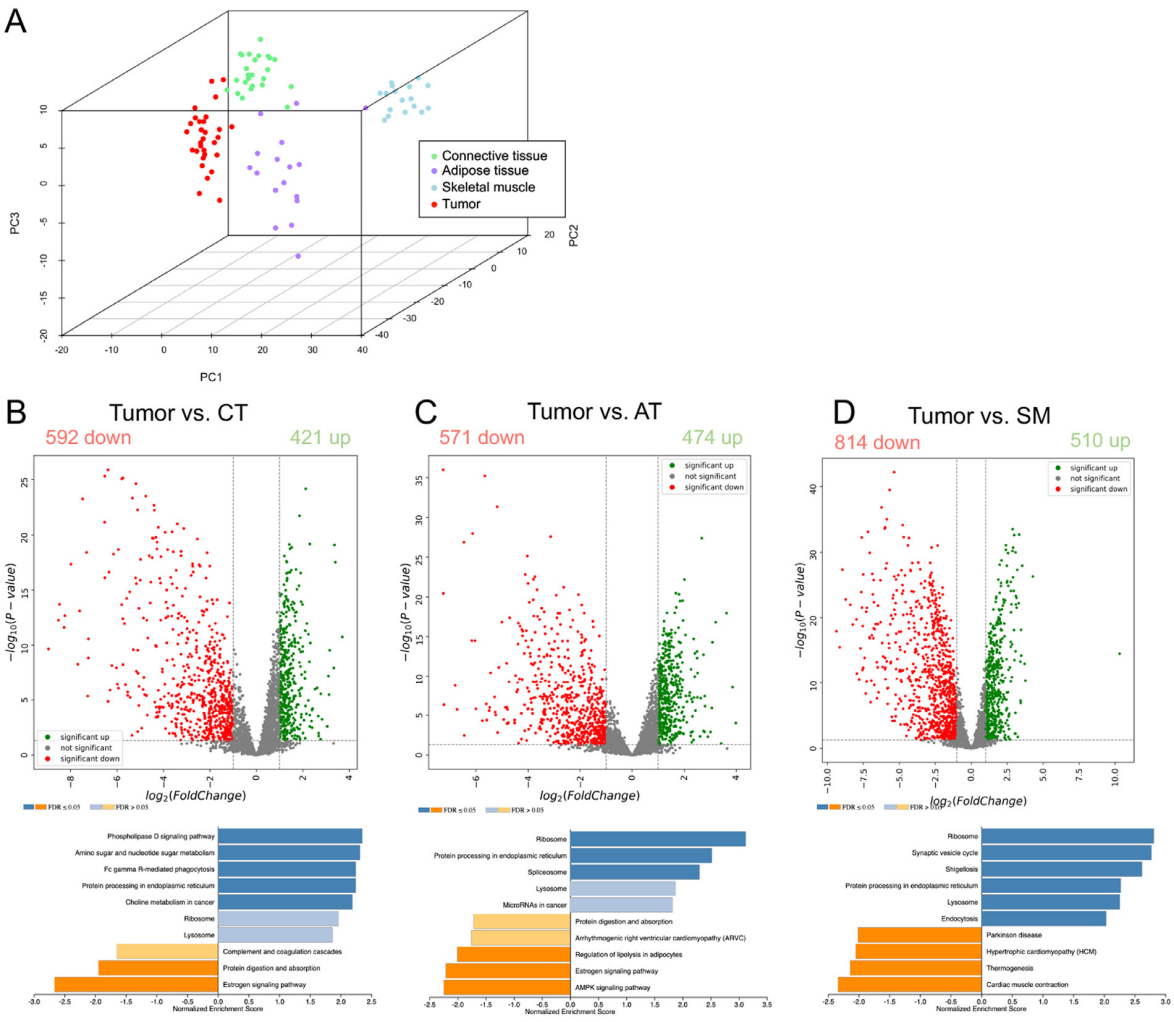
Proteomic analysis of FSA and surrounding PTT reveals strong expression differences between tissues. A) 3D-Principal Component Analysis plot of proteins detected in tumor and each PTT from 30 canine FSA. PCA was performed using all identified proteins. B-D) Volcano plots and Gene Set Enrichment analysis (GSEA) using KEGG pathways highlighting proteins that are differentially expressed between tumor and each PTT (red = significantly down in tumor, green = significantly up in tumor compared to respective PTT). Cutoff values for significance (p-value 0.05, log2(FC) = 1) are indicated using grey dashed lines. P-values were calculated using ANOVA; The volcano highlights proteins that are differently expressed between tumor tissue and B) connective tissue, C) adipose tissue, or D) skeletal muscle. GSEA for the individual comparisons are placed directly below the corresponding volcano plots.

Principal component analysis using all identified proteins revealed a clear separation between the four tissue types within the first three principal components (Fig. 2A). This highlights the distinction between the different tissue types as the major source of variability in the data and supports the validity of our approach to specifically analyze subsections of tumor and PTT from FFPE tissue using LCM-LC-MS/MS. Differential expression analysis of the proteins expressed in the various tissues using an adjusted p-value of 0.05 and log2 fold-change of >1 as cut-offs revealed the following. 1’013 proteins were significantly deregulated in tumor compared to CT, of which 421 were up- and 592 were down-regulated (Fig. 2B). With respect to AT, 1’045 proteins were significantly changing, with 474 proteins up- and 571 that were downregulated in tumor tissue (Fig. 2C). Compared to SM, 1’324 proteins were significantly changed in tumor, with 510 proteins that increased and 814 proteins that decreased in the tumor (Fig. 2D). Hence, FSA tumor cells display a clearly distinct protein expression signature compared to the three unaffected PTTs.

Gene set enrichment analysis (GSEA) of expression changes between tumor and the individual PTTs using KEGG pathways revealed the following. When comparing Tumor with CT, tumor tissue showed a significant enrichment of Phosopholipase D signaling pathway, Amino sugar and nucleotide sugar metabolism, Fc gamma R-mediated phagocytosis, Protein processing in the endoplasmic reticulum and choline metabolism in cancer, while CT was characterized by Estrogen signaling pathway and protein digestion and absorption (Fig. 2B, bottom). Comparison with AT resulted in ribosome, protein processing in endoplasmic reticulum and spliceosome to be enriched in tumor, while AT showed AMPK signaling pathway, Estrogen signaling pathway and regulation of lipolysis in adipocytes as the most enriched pathways (Fig. 2C, bottom). Finally, comparison of tumor with SM revealed an accumulation in ribosome, synaptic vesicle cycle, shigellosis, protein processing in endoplasmic reticulum, lysosome and endocytosis in tumor tissue, while cardiac muscle contraction, thermogenesis, hypertrophic cardiomyopathy and Parkinson disease dominated in SM (Fig. 2D, bottom). In summary, this is the first dataset to describe detailed proteomic changes between tumor cells and clinically relevant matched PTT in spontaneous FSA of any species.

### Identification of expression differences between tumor cells and PTT

**Fig. 3:**
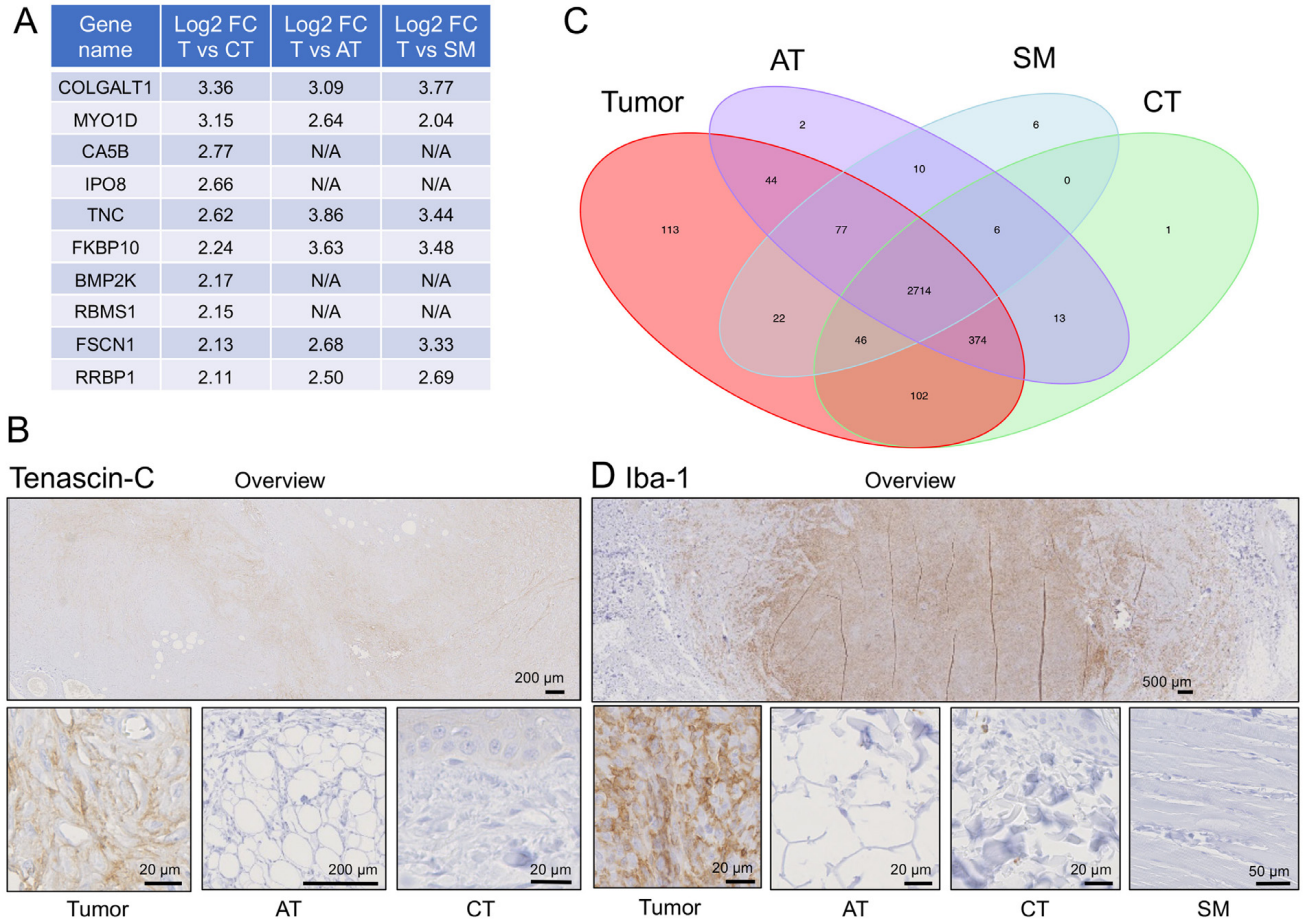
Identification of proteins that strongly differentiate FSA tumor tissue from all three surrounding PTTs. A) Proteins highly overexpressed in tumor tissue compared to all three PTTs (log2 fold-change ≥ 2, p-value < 0.05). N/A = not applicable, as protein was not detected in the respective tissue type. B) Representative image of immunohistochemical staining for Tenascin-C in a case of FSA (case #11). Tumor tissue is strongly staining, while staining in AT and CT is negligible or absent. C) Venn diagram showing all detected proteins per tissue type. 113 proteins were uniquely detected in tumor tissue. D) Representative image of immunohistochemical staining for Iba-1 in a case of FSA (case #19). Tumor tissue is strongly staining, while staining in AT and CT is negligible or absent.

To identify what exactly differentiates tumor cells from the surrounding PTT, we directly compared the four tissue types. 2’610 different proteins were found in 90% (27 cases out of 30) of all tumor samples and in at least one of the surrounding PTTs, and 2’275 of these were significantly differentially expressed between tumor and PTT (p-value 0.05) (Supplementary Table 1). Filtering for proteins using a log2 fold-change >2 and a p-value <0.05 for tumor/PTT revealed 10 proteins to be expressed much higher in tumor than in all of the surrounding three PTTs together (Fig. 3A). Among these, Tenascin-C (TNC) showed the highest consistent log2 fold-change, and verification by immunohistochemistry confirmed a marked increase in Tenascin-C protein levels in tumor compared to PTT (Fig. 3B).

113 proteins were exclusively detected in tumor tissue, whereas 2 were only present in AT, 6 in SM and 1 in CT (Fig. 3C). Of the 113 proteins restricted to tumor tissue, 25 could be found in 80% of all tumor samples and 5 were present in 90% or more (Table 2 and Supplementary Table 2). The top 5 proteins exclusively detected in tumor in ≥ 90% of the cases included CD68 (a macrophage marker), Optineurin (OPTN), Nuclear receptor coactivator 5 (NCOA5), RAP1GDS1 (Rap1 GTPase-GDP dissociation stimulator 1) and Stromal cell derived factor 2 like 1 (SDF2L1). In the absence of a CD68 antibody suitable for immunohistochemistry in canine tissue, we verified the presence of macrophages in tumor tissue using Iba-1, a marker for macrophages in dogs (Fig. 3D).

**Table 2:** **25 Proteins exclusively detected in FSA tumor tissue in  ≥ 80% of all samples.** # of tumors = number of tumor samples (out of 30 patients in total), in which the protein was detected.

Protein Accession	Protein description	Gene	# of tumors
F1P7M2	CD68 molecule	CD68	29
E2RBP8	Optineurin	OPTN	28
E2RL11	Nuclear receptor coactivator 5	NCOA5	28
E2RPS7	Rap1 GTPase-GDP dissociation stimulator 1	RAP1GDS1	28
E2RJV6	Stromal cell derived factor 2 like 1	SDF2L1	27
F1PIB1	Dishevelled segment polarity protein 3	DVL3	26
F1PSD7	Receptor tyrosine kinase like orphan receptor 2	ROR2	26
F1Q1H6	Integrin beta	ITGB2	26
E2QSF3	Spermatogenesis associated serine rich 2	SPATS2	25
E2R427	Rho guanine nucleotide exchange factor 7	ARHGEF7	25
E2RE97	Numb-like protein	COQ8B	25
E2RJX4	RAC-PK-alpha	AKT1	25
E2RPH0	Nudix hydrolase 9	NUDT9	25
F1PKM3	Procollagen-proline 4-dioxygenase	P4HA3	25
F1PMS0	LRR binding FLII interacting protein 1	LRRFIP1	25
F1PVD9	PYM homolog 1, exon junction complex associated factor	PYM1	25
J9P1M8	Nucleoporin NUP53	NUP35	25
E2R8E5	Serine and arginine rich splicing factor 5	SRSF5	24
E2RMM3	Microfibril associated protein 1	MFAP1	24
E2RMX1	Transmembrane protein 263	TMEM263	24
F1P9Z5	Scavenger receptor class A member 3	SCARA3	24
F1PA99	Solute carrier family 35 member B2	SLC35B2	24
F1PRW2	C5a anaphylatoxin chemotactic receptor	C5AR1	24
F1Q034	RING-type E3 ubiquitin transferase	ZNF598	24
Q38JA9	Caspase 8	CASP8	24

Hence, while FSA tumor tissue shares a lot of proteins with the surrounding AT, SM and CT, there are a number of proteins that are either strongly upregulated in or restricted to neoplastic tissue and could thus potentially serve to differentiate tumor from PTT.

### FSA tumor tissue is characterized by activated MYC and EIF2 signaling and a strong decrease in oxidative phosphorylation

**Fig. 4:**
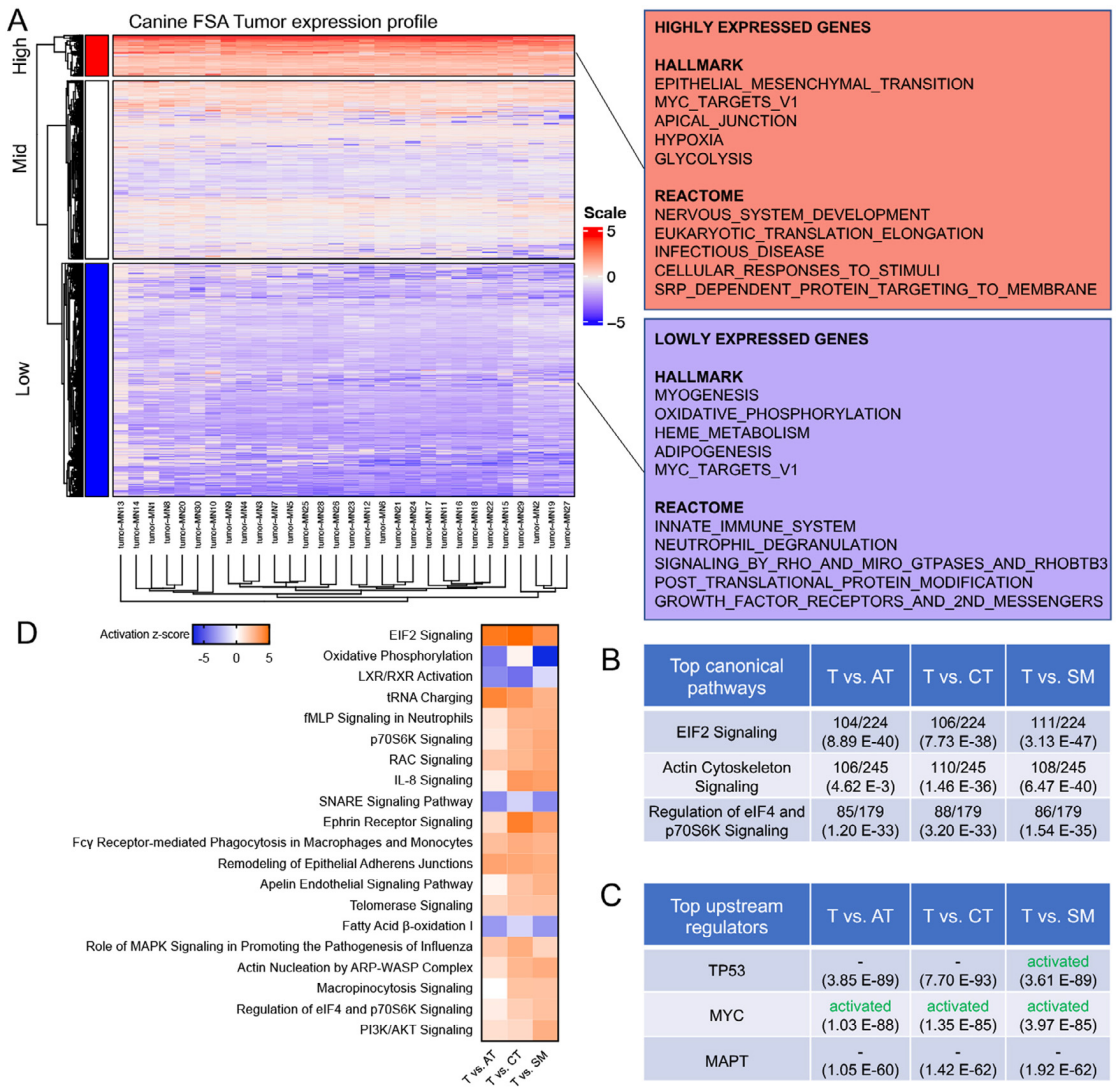
Molecular features of canine FSA. A) (left) Heatmap of all cases using all proteins showing highly homogeneous protein expression across all FSA cases. (right) GSEA using HALLMARK and REACTOME pathway databases of highly (top) and lowly (bottom) expressed proteins in FSA. B) Top canonical pathways detected in T vs AT, CT and SM using Ingenuity pathway analysis (IPA). Values on the top of a cell indicate how many targets were detected in the dataset/how many targets are attributed to the respective dataset, and values in brackets represent p-values. C) Top upstream regulators detected in T vs AT, CT and SM using Ingenuity pathway analysis (IPA). The top half of a cell indicates the predicted activation status, and values in brackets represent p-values. D) Activation status of top 20 canonical pathways differentially regulated between T vs AT, CT and SM.

To further explore the molecular features of FSA, we next analyzed the tumor tissue in more detail. Protein expression profiles were very consistent throughout all 30 tumor samples, suggesting that canine FSA represent a highly homogeneous entity on a molecular level (Fig. 4A). GSEA of the highly expressed genes using HALLMARK gene sets and the REACTOME pathway database revealed an enrichment in epithelial to mesenchymal transition, Myc targets, apical junction, hypoxia and glycolysis as well as nervous system development, eukaryotic translation elongation, infectious disease, cellular responses to stimuli and Srp-dependent protein targeting to membrane (Fig. 4A). In contrast, the lowly expressed genes featured myogenesis, oxidative phosphorylation, heme metabolism, adipogenesis and Myc targets as well as innate immune system, neutrophil degranulation, signaling by Rho and Miro GTPases and RHOBTB3, post-translational protein modification and growth factor receptors and 2^nd^ messengers (Fig. 4A). Additionally, we analysed the expression differences between tumor and PTT using QIAGEN Ingenuity Pathway Analysis (QIAGEN Inc., https://digitalinsights.qiagen.com/IPA). The top three canonical pathways detected for targets were identical between all juxtapositions of tumor vs individual PTT: EIF2 signaling, actin cytoskeleton signalling and regulation of eIF4 and p70S6K signaling (Fig. 4B). Likewise, the same top three upstream regulators were identified across all three comparisons and encompassed TP53 and MYC, both of which were predicted as activated in tumor cells compared to PTT as well as MAPT (Fig. 4C). Finally, analysis of the activation status of canonical pathways (taking into account targets differentially regulated between T and PTT with a p-value ≤0.05) revealed a strong activation of EIF2-signaling, tRNA charging, signalling in neutrophils, macrophages and monocytes, p70S6K signalling, RAC signalling, PI3K/Akt signalling and others (Fig. 4D). In contrast, the most strongly suppressed pathways were related to oxidative phosphorylation, LXR/RXR activation, SNARE signaling and fatty acid β-oxidation.

Hence, canine FSA are characterized by oncogenic signaling driven strongly by MYC and possibly TP53, hyperactive EIF2 and immune-related signaling as well as strongly decreased oxidative phosphorylation, coinciding with glycolysis and hypoxia, and oxidative lipid metabolism.

### Canine FSA display a high grade of molecular homology with human soft-tissue sarcomas

**Fig. 5:**
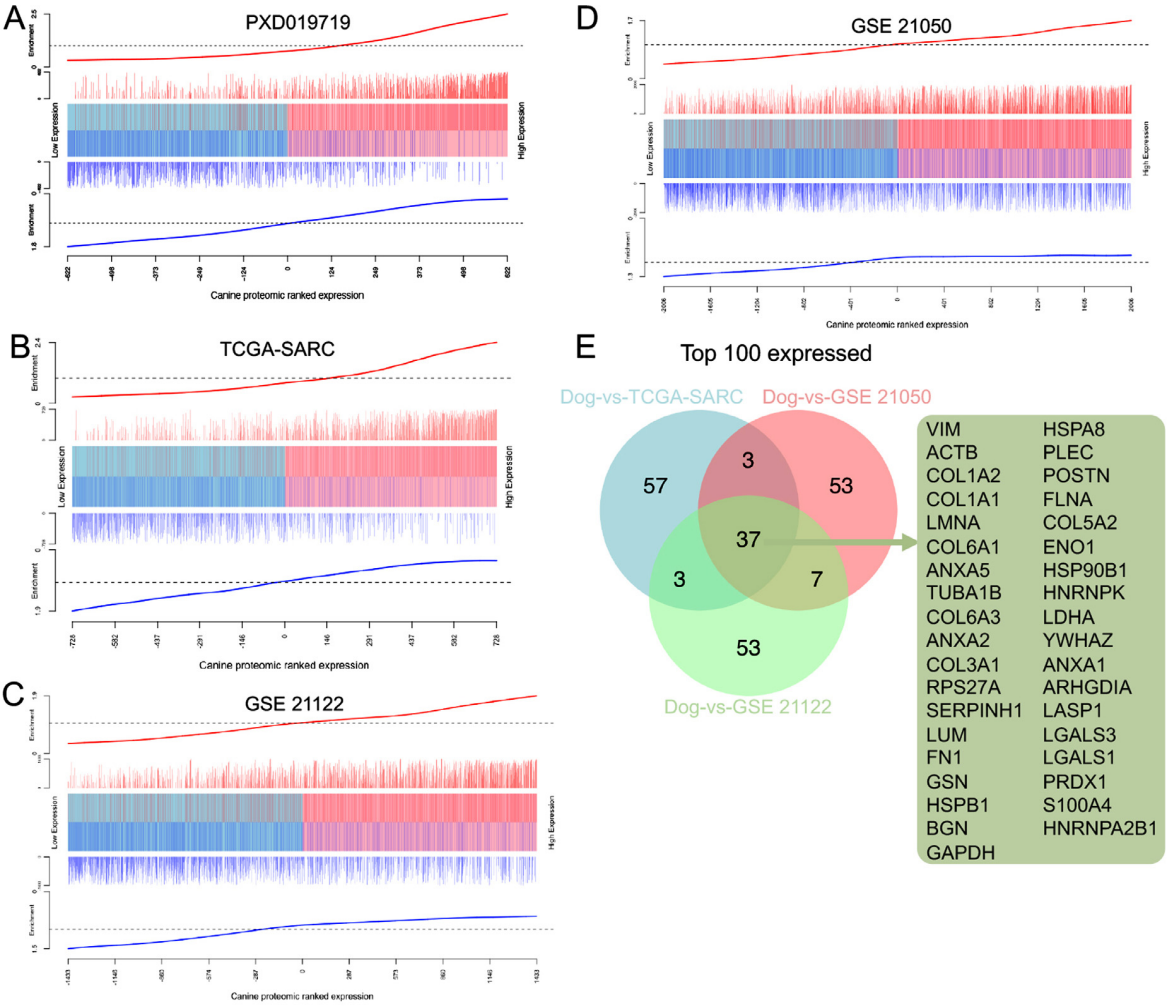
Canine FSA displays significant molecular homology with human STS. A)– D) Competitive gene set testing to compare canine FSA to human STS datasets A) PXD019719, B) TCGA-SARC, C) GSE 21122, and D) GSE 21050. GSEA-like running sum statistic depicting the location of human STS proteins on a ranked list of genes in our canine FSA dataset. Upregulated genes identified in human STS are indicated as red vertical bars, downregulated genes identified in human STS are indicated as blue vertical bars. E) Venn diagram of top 100 overexpressed targets from B – D. Gene list on the right shows all targets found in all three of these comparisons.

Given that canine STS have been suggested to be potentially useful models for the human condition, we next wanted to exploit whether and to what degree canine FSA can be compared to the human STS on a molecular level. Due to the lack of available datasets that have specifically analyzed human FSA on a proteomic level, we set out to compare our dataset with published proteomic data of human STS in general. We took advantage of a proteomic dataset of a cohort of 36 STS patients including four histological subtypes (leiomyosarcoma, synovial sarcoma, undifferentiated pleomorphic sarcoma and dedifferentiated liposarcoma [28]). We postulated that if canine and human tumors were to share a high level of molecular homology, differentially expressed proteins in canine FSA should exhibit a similar expression pattern in the human STS dataset. To test this hypothesis, we first identified all genes that were present in both the canine and the human dataset and ranked these 1’244 proteins in the canine dataset based on expression from low to high. We then assessed the enrichment of proteins in the human dataset in this ranked gene list. Remarkably, we found the upregulated subset of the human STS signature (red vertical bars) to be strongly enriched on the right-hand side of the ranked gene list and hence with the highly expressed genes in canine FSA (Fig. 5A). In contrast, the downregulated subset of the human STS signature (blue vertical bars) was clearly enriched on the left-hand side, which is associated with proteins that have low expression in canine FSA. Correlation analysis suggested these results to be highly significant (p < 0.0001).

Due to the very limited availability of MS-based proteomic data on human STS [31], we further took advantage of the extensive RNAseq dataset on human STS of connective, subcutaneous and other soft tissues generated by TCGA (TCGA-SARC [32]). This dataset includes a total of 117 tumors, including 36 fibromatous, 9 lipomatous and 27 myomatous neoplasms, 6 nerve sheath tumors, 30 soft tissue tumors and sarcomas not otherwise specified as well as 9 synovial-like neoplasms. 1’457 common targets were identified between the two datasets (Fig. 5B). Similar to the proteomic dataset, enrichment of the upregulated canine FSA signature significantly overlapped with the most upregulated human genes and vice versa for the downregulated genes (Fig. 5B; correlation p < 0.0001). A very similar picture emerged when running the comparison against two microarray-based cohorts GSE21122 (150 cases encompassing 7 major subtypes [33]; 2866 common targets (Fig. 5C); correlation p < 0.0001) and GSE21050 (310 cases, with 136 undifferentiated sarcomas, 85 leiomyosarcomas, 62 dedifferentiated liposarcomas, and 27 other subtypes [34]) 4013 common targets, Fig. 5D; correlation p < 0.0001). Finally, of the top 100 expressed common targets between the individual comparisons from Fig. 5B-D, 37 targets were shared across all the three comparisons including VIM, ACTB, COL1A2, COL1A1, LMNA and others (Fig. 5E).

In conclusion, these results clearly demonstrate the presence of significant molecular homology between canine FSA and human STS.

## Discussion

Despite the progress made in the understanding of the genetic drivers of tumor formation over the last decades, our molecular understanding of STS at the protein level is still in its infancy [31,35]. Proteomic analysis of tumor tissue offers an exciting tool to complement genetic and translational data and could play an important part in understanding the molecular mechanisms driving STS development and progression, identifying potential biomarkers predicting response to treatment and identification of novel therapeutic targets [31,35]. The latter is among the most important strengths of proteomics, as proteins represent the largest group of druggable targets available. Furthermore, with the help of proteomics, proteins and their structure, interactions, modifications and physiological function can be analyzed and taken together, the proteome reflects the functional information of genes [36]. Unfortunately, the rarity and heterogeneity of STS make them a challenging entity to investigate [31,35]. As this translates to poor evolution of STS treatment, it represents an unmet need that needs to be addressed. So far, only few proteomic studies are available for STS. Among these, only few have specifically investigated some of the more frequent subtypes, while others have included integrative analysis of multiple histologic subtypes to reach statistically significant cohort sizes [35]. As a consequence, molecular data for ultra-rare subtypes of STS is extremely scarce, and to the best of our knowledge there are currently no proteomic studies that investigate the proteomic landscape of naturally occurring human adult FSA - neither subtype specific nor embedded within a larger cohort. For canine FSA, only two studies have attempted a more detailed look into underlying molecular changes: one focused on a chromosomal translocation involving the CDKN2B locus, and the other compared 5 cases of PNST to 5 FSA by microarray to find subtype-specific differences [37,38]. Proteomic data on canine FSA is currently inexistent.

The present study was aimed at providing detailed insight into proteomic changes between FSA and its surrounding normal tissue of 30 canine patients. We harnessed a powerful approach incorporating LC-MS/MS-based proteomic analysis of microdissected tumor and PTT. Overall, our results demonstrate that FSA can be clearly differentiated from its PTT based on protein expression, and that canine FSA shares homology with available proteomic data of various human STS subtypes. These results will contribute to refined understanding of FSA biology in general, underline the potential of comparative studies between human and canine FSA, and have the potential to reveal novel diagnostic markers and therapeutic targets.

Our data revealed several interesting insights into canine FSA. COLGALT1, MYO1D, CA5B, IPO8, TNC, FKBP10, BMP2K, RBMS1, FSCN1 and RRBP1 are all highly overexpressed in tumor compared to all of the other three PTT analyzed (Fig. 3A). Among these targets, TNC, FSCN1 and RRBP1 have been implicated in STS pathogenesis. TNC is an extracellular matrix protein implicated in guidance of migrating neurons as well as endothelial cells, and in progression of many different types of tumors [39]. Interestingly, it also overexpressed in chondrosarcoma, Ewing sarcoma and dermatofibrosarcoma protuberans and known to promote cancer cell survival and migration as well as metastasis and malignancy therein [40], [41], [42], [43]. Furthermore, TNC has been advocated as a potential target in STS treatment, and TNC specific ligands are available for experimental use [44]. FSCN1, an actin-binding protein with important functions in cell motility and migration, has been found to play an important role in development of osteosarcoma and chondrosarcoma [45,46]. RRBP1, a ribosome receptor that mediates interaction between ribosomes and the endoplasmic reticulum, has been detected as RRBP1-ALK fusion protein in epitheloid inflammatory myofibroblastic sarcoma [47]. The extent of the involvement of the remaining targets on development of FSA remains to be investigated.

We identified 25 proteins that were exclusively found in at least 80% of tumors but none of the PTT (Table 2). It will be highly interesting to address the suitability of these markers as tools to support molecular diagnosis or targeted therapy of FSA. Among the proteins exclusively detected in tumor, CD68 as a macrophage marker stands out. Indeed, in one analysis including 38 canine STS arising in limbs and trunc, between 6% - 62% of cells in a given microscopic field were shown to be macrophages [48]. Furthermore, the number of macrophages positively correlated with histologic grade of the tumor and with the number of mitoses found in the tissue, suggesting macrophages to be an important feature driving tumor grade. Similarly, CD74-positive macrophages have been found to promote tumor growth in a mouse model of undifferentiated pleomorphic sarcoma [49]. In human STS, UPS and Myxofibrosarcomas were found to have the highest median macrophage score of all sarcoma types [50], and a high number of CD68+ macrophages are discussed as negative prognostic factors for a series of STS, including Osteosarcoma and Ewing Sarcoma (reviewed in [51]). Based on previous findings, targeting tumor associated macrophages (TAM) for therapeutic reasons has been considered promising, yet challenging [52]. Our results underline, that TAMs might also be an interesting target in canine FSA. However, a more concrete understanding of the exact TAM phenotype and function in FSA are needed to unlock this potential treatment option in the future. Likewise, we identified ROR2 among the tumor-exclusive proteins. ROR2 is a receptor tyrosine kinase involved in WNT-5a signaling, has been described as a prognostic biomarker as well as a potential therapeutic target in gastrointestinal stromal tumors and leiomyosarcomas [53], and its expression correlates with disease severity in osteosarcoma [54]. It has been identified to be a regulator of osteosarcoma cell invasiveness, presenting a potential therapeutic target in osteosarcoma [55,56]. The role of ROR2 in canine FSA remains to be elucidated in more detail.

On the level of signaling pathways, predicted activation of EIF2-, Myc-, TP53- and PI3K/Akt-signaling are intriguing (Fig. 4B-D). EIF2 is a translation initiation factor that integrates a diverse array of stress-related signals including hypoxia, oxidative stress and nutrient starvation to trigger activation of the so-called integrative stress response, which in turn mediates cell survival under adverse circumstances [57,58]. Given the strong emergence of a hypoxic signature and a strong decrease in oxidative phosphorylation and fatty acid oxidation (Fig. 4A and D), it is conceivable that hypoxia is the strongest driver of this signature in our dataset. Importantly, misregulation of EIF2 has been associated with various types of tumors and suggested to be an interesting therapeutic target [57]. Moreover, Myc signaling has been described to activate EIF2 signaling in various tumors (reviewed in [59]), which is in line with activated Myc signaling detected in our tumor samples (Fig. 4C). High expression of Myc has been described as a prognostic biomarker in a variety of human STS (e.g. leiomyosarcomas, osteosarcomas and liposarcomas) and a high frequency of MYC gene amplification is associated with radiation-induced sarcomas [60], [61], [62], [63]. In canine osteosarcomas, mutations in both TP53 and Myc have been described, but neither prognostic nor therapeutic value of this finding are currently known [64]. A more profound understanding of the molecular basis of MYC activation in our dataset might reveal further vulnerabilities of canine FSA in the future. Similarly, the PI3K/Akt-pathway has been shown to be significantly associated with more aggressive STS and to be involved in maintenance of cancer stem cells in sarcomas, meriting future detailed analysis [65,66].

Finally, we present evidence that canine FSA share extensive molecular homology with human STS (Fig. 5). Given the sparsity of molecular data on human FSA, it will certainly be highly interesting to compare canine FSA to its human counterpart to further delineate similarities between the disease in the two species in the future. Independent from these missing direct comparisons, and although a one fits all approach is unlikely to be successful for an entity as heterogeneous as STS, our findings underline the translational value of dogs in the context of STS research. The documented homologies of canine FSA with the most frequent STS subtypes in humans could present a key to a deeper understanding of these conditions in both species in the future. As STS is 10-100 times more frequent in dogs, it is mandatory to further investigate above mentioned molecular homologies and to test the response to new therapeutics against the identified most common targets.

Collectively, the results presented herein will serve as starting point for further studies on FSA – both human as well as canine – to better understand the biology driving these tumors and also to develop specific diagnostic and therapeutic approaches to benefit patients from both species.

### Data and code availability

The mass spectrometry proteomics data have been deposited to the ProteomeXchange Consortium via the PRIDE [67] partner repository with the dataset identifier PXD029338. All other data supporting our findings is contained in the manuscript and in the supplementary figures and tables.

## Author contributions

M.C.N and E.M. planned and initiated the study. E.B. and Z.M. performed LCM. A.P., E.B. and E.M. performed data analysis. F.G. is nationally certified pathologist and performed and supervised choice of clinical cases and areas to be isolated by LCM. F.G. performed immunohistochemistry. L.K. analyzed all samples by LC-MS/MS and W.W. performed initial bioinformatic analysis of the LC-MS/MS data. D.M. provided several of the included cases for analysis. E.M. and M.C.N. were responsible for study design, supervision, data analysis and funding. A.P., E.B. and E.M. wrote the first draft of the manuscript. All authors read, contributed to, and approved the final manuscript.

## Declaration of interests

The authors declare no competing interests.
